# The impact of adjusting for baseline in pharmacogenomic genome-wide association studies of quantitative change

**DOI:** 10.1038/s41525-019-0109-4

**Published:** 2020-01-16

**Authors:** Akinyemi Oni-Orisan, Tanushree Haldar, Dilrini K. Ranatunga, Marisa W. Medina, Catherine Schaefer, Ronald M. Krauss, Carlos Iribarren, Neil Risch, Thomas J. Hoffmann

**Affiliations:** 1 Department of Clinical Pharmacy, University of California, San Francisco, CA 94143 USA; 2 Institute for Human Genetics, University of California, San Francisco, CA 94143 USA; 3 Division of Research, Kaiser Permanente Northern California, Oakland, CA 94612 USA; 4 Children’s Hospital Oakland Research Institute, Oakland, CA 94609 USA; 5 Department of Medicine, University of California, San Francisco, CA 94143 USA; 6 Department of Epidemiology and Biostatistics, University of California, San Francisco, CA 94158 USA

**Keywords:** Pharmacogenetics, Genetics research, Dyslipidaemias

## Abstract

In pharmacogenomic studies of quantitative change, any association between genetic variants and the pretreatment (baseline) measurement can bias the estimate of effect between those variants and drug response. A putative solution is to adjust for baseline. We conducted a series of genome-wide association studies (GWASs) for low-density lipoprotein cholesterol (LDL-C) response to statin therapy in 34,874 participants of the Genetic Epidemiology Research on Adult Health and Aging (GERA) cohort as a case study to investigate the impact of baseline adjustment on results generated from pharmacogenomic studies of quantitative change. Across phenotypes of statin-induced LDL-C change, baseline adjustment identified variants from six loci meeting genome-wide significance (*SORT/CELSR2/PSRC1, LPA, SLCO1B1, APOE, APOB*, and *SMARCA4*/*LDLR*). In contrast, baseline-unadjusted analyses yielded variants from three loci meeting the criteria for genome-wide significance (*LPA*, *APOE*, and *SLCO1B1*). A genome-wide heterogeneity test of baseline versus statin on-treatment LDL-C levels was performed as the definitive test for the true effect of genetic variants on statin-induced LDL-C change. These findings were generally consistent with the models not adjusting for baseline signifying that genome-wide significant hits generated only from baseline-adjusted analyses (*SORT/CELSR2/PSRC1, APOB, SMARCA4*/*LDLR*) were likely biased. We then comprehensively reviewed published GWASs of drug-induced quantitative change and discovered that more than half (59%) inappropriately adjusted for baseline. Altogether, we demonstrate that (1) baseline adjustment introduces bias in pharmacogenomic studies of quantitative change and (2) this erroneous methodology is highly prevalent. We conclude that it is critical to avoid this common statistical approach in future pharmacogenomic studies of quantitative change.

## Introduction

Pharmacogenomic studies of continuous (quantitative) phenotypes most commonly identify genetic determinants of the change between pretreatment (baseline) and on-treatment levels from the administration of a therapeutic drug intervention. This approach has improved statistical power in detecting a genetic effect over using dichotomous outcomes (i.e., case-control design), especially when the dichotomous outcome is rare.^1^

A critical objective of this type of analysis is to identify genetic effects on drug-induced changes independent of the baseline value, especially when it is known that the baseline value itself has a strong genetic component. Any association between genetic variants and the baseline measurement can produce a false-positive association between that variant and the drug response phenotype. A putative solution is to add the baseline value as a covariate to the linear regression model. However, it has been documented in multiple studies of statistics and epidemiology that this analytical approach (adjusting for baseline) may introduce the bias that it seeks to prevent.^2–6^

Here, we present the results for a series of statin-induced low-density lipoprotein cholesterol (LDL-C) response genome-wide association studies (GWASs) as a case example illustrating that the bias introduced when adjusting for baseline also applies to pharmacogenomic analyses. We also report the results of a comprehensive literature search to determine the prevalence of adjusting for baseline in pharmacogenomic studies.

## Results

### Population and genetic characteristics

A total of 34,874 statin users from the GERA cohort met the criteria for inclusion. The study population was multiethnic (Supplementary Table 1). The median percent reduction in LDL-C from statin treatment was 35% (interquartile range (IQR), 24−45%). There were 17,708,023, 16,329,859, 8,984,232, and 10,307,784 variants that remained for analyses in White/European, Black/African, East Asian, and Hispanic/Latino participants, respectively. Of these, 13,250,765 variants were shared between two or more race/ethnicity groups: these were carried forward for the GWASs. There was no evidence of genomic inflation^7^ based on a genomic inflation factor (*λ*) value of 0.997 in the combined population (for baseline-unadjusted percent change in LDL-C from statin therapy). Likewise, the genomic inflation factor within each race/ethnicity group was 1.001, 1.001, 1.005, and 0.983 for White/European, Black/African, East Asian, and Hispanic/Latino participants, respectively.

### The impact of phenotype and baseline-adjustment in GWASs of statin LDL-C response

**Fig. 1:**
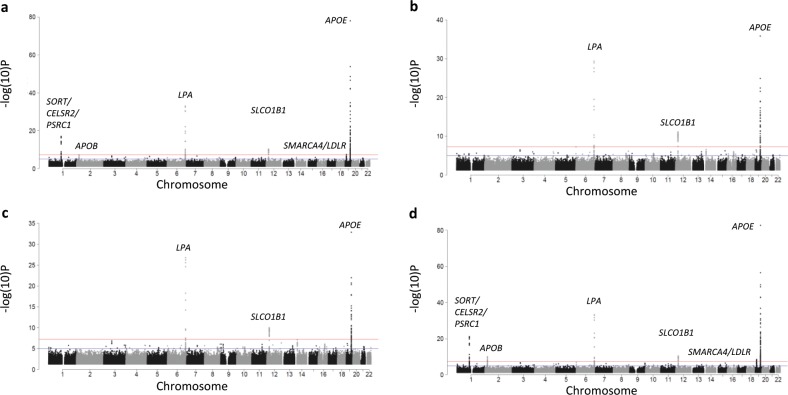
Manhattan plots for a series of genome-wide association studies (GWAS) of statin-induced low-density lipoprotein cholesterol (LDL-C) response, highlighting key chromosomal regions. A GWAS for baseline-adjusted difference of natural log-transformed LDL-C levels yielded six significant loci (**a**), whereas a GWAS of baseline-unadjusted statin-induced percent LDL-C lowering yielded three loci that met genome-wide significance (**b**). Consistent with the impact of baseline-adjustment on results, baseline-unadjusted difference of natural log-transformed LDL-C levels (**c**) and baseline-adjusted statin-induced percent LDL-C lowering (**d**) yielded three and six genome-wide significant loci, respectively. All tests were two-sided.

The GWAS of statin LDL-C response using the Postmus et al. definition (the difference of natural log-transformed baseline and on-treatment LDL-C levels adjusted for the natural log-transformed baseline level) revealed variants from six loci that met genome-wide significance (*SORT/CELSR2/PSRC1, LPA, SLCO1B1, APOE, APOB*, and *SMARCA4*/*LDLR*; Fig. 1a, Table 1). In contrast, the GWAS of statin LDL-C response using our previous definition (statin-induced LDL-C percent lowering without adjustment for baseline LDL-C) yielded only three loci that met genome-wide significance: *LPA*, *SLCO1B1*, and *APOE* (Fig. 1b, Table 1). It was unknown if the phenotype itself, baseline-adjustment, or both were impacting this discrepancy in results (i.e., the discrepant count of significant genome-wide loci). When we altered the Postmus et al. definition so that baseline was not included as a covariate (the difference of natural log-transformed baseline and on-treatment LDL-C levels without adjustment for baseline), we identified only the three same loci as statin-induced percent LDL-C lowering without baseline adjustment (*LPA*, *SLCO1B1*, and *APOE;* Fig. 1c). This suggested that baseline-adjustment was impacting the number of significant genome-wide loci. The GWAS of statin-induced percent LDL-C lowering with adjustment for baseline LDL-C identified the same six loci as the Postmus et al. definition (*SORT/CELSR2/PSRC1, LPA, SLCO1B1, APOE, APOB, SMARCA4*/*LDLR*; Fig. 1d), further confirming the impact of baseline-adjustment on results. Taken together, when baseline LDL-C was included as a covariate in the GWAS regression model, six loci met genome-wide significance; when baseline was not included as a covariate, three loci met the threshold of genome-wide significance. Effect size magnitude and direction did not vary significantly by race/ethnicity (Table 1, Supplementary Tables 2–5).

**Table 1:** All six lead variants from the genome-wide significant meta-analysis loci for baseline-adjusted difference of natural log-transformed statin on- and baseline low-density lipoprotein cholesterol levels by statin response variable in combined race/ethnicity groups (*N* = 34,874).

Gene	SNP	CHR	BP	Minor allele	MAF	Statin response variable^a^	Beta (SE)^b^	*P* value	Cochrane’s Q statistic *P* value^c^	I^2 heterogeneity index (0–100)^c^
*SORT1*	rs7528419	1	109817192	G	0.198	Baseline-adjusted	−0.019 (0.002)	9.55E-18	0.944	0
						Baseline-unadjusted	−0.046 (0.010)	3.59E-06^d^	0.496	0
						Interaction		0.009^d^		
						Baseline only	−0.035 (0.002)	1.57E-54	0.352	8
*APOB*	rs1713222	2	21271323	A	0.145	Baseline-adjusted	−0.013 (0.002)	4.68E-08	0.255	26
						Baseline-unadjusted	−0.031 (0.011)	5.05E-03^d^	0.455	0
						Interaction		0.176^d^		
						Baseline only	−0.027 (0.003)	1.09E-26	0.670	0
*LPA*	rs10455872	6	161010118	G	0.068	Baseline-adjusted	0.042 (0.003)	1.01E-33	0.128	51
						Baseline-unadjusted	0.179 (0.016)	4.24E-30	0.160	45
						Interaction		1.21E-11		
						Baseline only	0.008 (0.003)	0.019^d^	0.593	0
*SLC01B1*	rs58310495	12	21357711	T	0.179	Baseline-adjusted	0.015 (0.002)	4.58E-11	0.225	31
						Baseline-unadjusted	0.069 (0.010)	7.48E-12	0.247	28
						Interaction		5.83E-04^d^		
						Baseline only	−0.001 (0.002)	0.622^d^	0.489	0
*SMARCA4/LDLR*	rs67337506	19	11207982	C	0.250	Baseline-adjusted	−0.012 (0.002)	3.08E-08	0.602	0
						Baseline-unadjusted	−0.033 (0.010)	8.41E-04^d^	0.609	0
						Interaction		0.043^d^		
						Baseline only	−0.017 (0.002)	1.26E-13	0.162	42
*APOE*	rs7412	19	45412079	T	0.070	Baseline-adjusted	−0.068 (0.004)	1.08E-78	0.35	5
						Baseline-unadjusted	−0.202 (0.016)	1.46E-36	0.229	32
						Interaction		7.89E-19		
						Baseline only	−0.086 (0.004)	1.67E-118	0.068	63

*BP* base pair, *CHR* chromosome, *MAF* minor allele frequency, *SE* standard error, *SNP* single nucleotide polymorphism

^a^Baseline-adjusted difference of natural log-transformed statin on- and baseline low-density lipoprotein cholesterol levels, baseline-unadjusted statin-induced percent low-density lipoprotein cholesterol lowering, interaction of natural log-transformed statin on- versus baseline low-density lipoprotein cholesterol levels, and natural log-transformed baseline low-density lipoprotein cholesterol level

^b^Fixed effects calculated with respect to the minor allele. A negative value indicates more intense statin LDL-C lowering

^c^Refers to the variation on results between race/ethnicity groups

^d^Not reaching genome-wide significance (*P* < 5E-08)

**Fig. 2:**
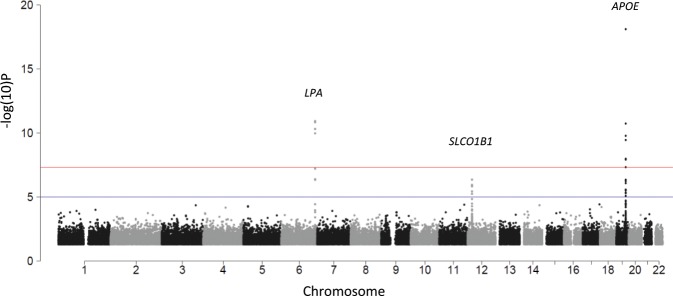
Manhattan plot for the genome-wide heterogeneity test of baseline versus statin on-treatment low-density lipoprotein cholesterol (LDL-C) levels. Results analyzing the interaction of genetic variants on statin LDL-C response revealed variants from two loci that met genome-wide significance and variants from one loci that met suggestive statistical significance. A Cochran’s Q test comparing baseline versus on-treatment betas was performed to test the gene−drug interaction of each variant. All tests were two-sided.

We then conducted an interaction test, a genome-wide heterogeneity test of statin baseline versus on-treatment LDL-C levels, as the definitive test for the true effect of genetic variants on statin-induced LDL-C change. Findings were generally consistent with the baseline-unadjusted models: variants from *LPA* and *APOE* met genome-wide significance and variants from *SLCO1B1* met suggestive significance (Fig. 2, Table 2).

**Table 2:** Lead variants of the significant loci from the genome-wide heterogeneity test of baseline versus statin on-treatment low-density lipoprotein cholesterol levels in combined race/ethnicity groups (*N* = 34,874).

Gene	SNP	CHR	BP	Minor allele	MAF	Baseline beta^a^	On-treatment beta^a^	*P* value	I^2 heterogeneity index (0–100)^b^
*LPA*	rs10455872	6	161010118	G	0.068	0.008 (0.004)	0.048 (0.004)	1.21E-11	98
*SLC01B1*	rs73079476	12	21343833	C	0.152	0.0004 (0.004)	0.016 (0.004)	4.49E-07^c^	96
*APOE*	rs7412	19	45412079	T	0.070	−0.086 (0.003)	−0.128 (0.003)	7.89E-19	99

*BP* base pair, *CHR* chromosome, *MAF* minor allele frequency, *SNP* single nucleotide polymorphism

^a^Fixed effects calculated with respect to the minor allele

^b^Refers to the variation on results between baseline versus statin on-treatment low-density lipoprotein cholesterol levels

^c^Suggestive of genome-wide significance (*P* < 1E-05)

Separate GWASs of baseline and on-treatment LDL-C levels each revealed multiple genome-wide associations as expected (Supplementary Figs. 1 and 2). Notably, among the three loci meeting genome-wide significance only when baseline was adjusted for (and not in the analyses of baseline-unadjusted phenotypes), all were also significantly associated with baseline LDL-C levels at the genome-wide level: *SORT/CELSR2/PSRC1, APOB, SMARCA4*/*LDLR* (Table 1). In contrast, among the three loci identified in the unadjusted analyses (*LPA, SLCO1B1, APOE*), only *APOE* was associated with baseline LDL-C levels (Table 1).

### Baseline adjustment in previous genome-wide pharmacogenomic studies of quantitative change

Among GWAS studies in the NHGRI-EBI GWAS Catalog, 59 included ≥1 quantitative drug response phenotype (using baseline and on-treatment measures) where covariates were added to the linear regression model (Supplementary Table 6). These studies investigated drug response to a variety of disease biomarkers including asthma, diabetes, dyslipidemia, hypertension, schizophrenia, depression, and others. Among the 59, 35 (59%) adjusted the drug-induced change phenotype for baseline values. At the time of the literature search, the year of publication for these studies ranged from 2009 to 2018. A majority of the studies (21 of 35; 60%) were published within the last 3 years from the time of literature search (2016 to 2018).

## Discussion

An important source of bias in studies of quantitative change is the potential impact of the baseline measurement on the change. In this report, we extend the work of previous studies on this topic to the field of pharmacogenomics through a series of genome-wide analyses. We demonstrate that the number of significant associations can be strongly influenced by baseline adjustment. We also suggest, through the results of a systematic literature search, that confusion exists surrounding baseline adjustment in recent pharmacogenomic studies of quantitative change.

An excellent paper that touched on this topic was published in 2008 (online) by McArdle and Whitcomb.^8^ In this publication, the authors used simulations with blood pressure measurements of the HAPI Heart Study (the mean, distribution, and measurement error of the blood pressures were simulated; observed measurements from the HAPI Heart Study were used to ensure the measurements were biologically plausible) and genotype data for loci in an area of chromosome 2q to demonstrate the bias introduced from adjusting for baseline in genetic association studies. However, at the time of that publication, there were less than a dozen pharmacogenomic GWASs published with only a few investigating phenotypes of quantitative change.^9^ In the current study, we use full-genome and real-world (not simulated) data to report the false-positives that appear as a result of baseline-adjustment in pharmacogenomic studies. Furthermore, now that a decade has passed since the McArdle and Whitcomb article (allowing time for the number of published pharmacogenomic GWASs to accumulate), we also report the prevalence of analyses performing this improper baseline-adjustment approach.

It turns out that a likely source of this bias is the “regression toward the mean” phenomenon.^2,3^ Briefly, baseline values should not necessarily impact the quantitative change. However, any measurement error in the baseline value will produce a false statistical correlation between the baseline and change.^6^ This is because any baseline measurement error will be negatively correlated with an observed change (e.g., baseline error in the positive direction from the true baseline measurement value will show an observed change in the negative direction: enhanced reduction if the true change causes a reduction in value or attenuated increase if the true change increases the value). The error in the second measurement will not produce changes of a great enough magnitude to balance out changes from errors in the baseline measurement. Accordingly, any association between a covariate in the model and the apparent baseline value will also falsely correlate with the quantitative change, since there is now an artificial statistical relationship between baseline and change. Thus the “regression toward the mean” in this case of quantitative change is related to the error for the second measurement, which is likely to be less extreme than the original baseline measurement error. This is the case whether the baseline error is at a positive or negative extreme. Importantly, measurement error must be present for this biasing to occur. However, all laboratory values inherently have some degree of analytical error. Thus, adjusting for baseline has the potential to bias data regardless of study, disease state, or phenotype.

In regards to the current study, we previously estimated overall measurement error in LDL-C values from the GERA cohort to be 34%.^10^ Since statin therapy generally produces large reductions in LDL-C levels, any error in baseline LDL-C levels in a positive direction (for example) from the true LDL-C value will tend to be associated with falsely larger reductions in LDL-C than in actuality. An error in the statin on-treatment LDL-C level that is also in the positive direction would have an opposite correlation on LDL-C change (compared to the aforementioned baseline LDL-C measurement error in the positive direction). But as stated above, extreme error in baseline levels is not balanced out by the error from the second measurement of LDL-C due to “regression toward the mean”. This imbalance results in a false correlation between baseline LDL-C levels and statin LDL-C response (i.e., a more positive apparent baseline level is correlated with larger statin LDL-C reduction and vice versa). Accordingly, the addition of baseline LDL-C to a regression model of statin LDL-C change will produce false correlations between any covariate that is truly associated with baseline (e.g., genetic variants) and the quantitative change. It should be mentioned that regardless of whether the response variable is in units of percent reduction from baseline (%) or absolute difference (mg/dL), this principle holds as our data shows (Supplementary Table 7).

In addition to the aforementioned large measurement error of LDL-C levels, it is established that untreated (baseline) LDL-C is highly heritable with several genome-wide predictors.^10^ Both features of LDL-C make GWASs of statin-induced LDL-C response a good case study to illustrate the impact of baseline adjustment in pharmacogenomic studies of quantitative change.

To demonstrate the impact of baseline adjustment, we used two regression models (either adjusting for baseline or not) for each of two statin-induced LDL-C change phenotypes. We found that adjusting for baseline led to more genome-wide associations compared to unadjusted models (6 versus 3 hits). Unlike the effect of baseline adjustment in the regression model, the specific phenotype (statin-induced percent LDL-C lowering vs. the difference of natural-log-transformed LDL-C levels) had no impact on results. We then performed a genome-wide heterogeneity study investigating the interaction of statin treatment on genetic variant LDL-C effects. These results mirrored our baseline-unadjusted findings confirming the appropriateness of not adjusting for baseline in our regression models. We believe that the extra genome-wide hits (*SORT1*, *APOB*, *SMARCA4*/*LDLR*) from the baseline-adjusted analyses are false-positives. All three of these loci were found to be strong predictors of baseline LDL-C in our current report as well as in previous studies.^10^

Ultimately, we identified three unconfounded genome-wide significant loci of statin-induced LDL-C response: *LPA*, *APOE*, *SLCO1B1*. These loci have been previously identified among the six past GWASs of statin LDL-C response.^11–16^ Specifically, multiple GWASs (including baseline-unadjusted analyses) have shown associations between *LPA* and *APOE* with statin response. Only one previous study (Postmus et al.) identified *SLCO1B1* as a genome-wide hit.^15^ As a meta-analysis (19 observational and RCT studies covering 40,914 European participants), this is the only previous GWAS that investigates genetic predictors of statin LDL-C lowering with a sample size that is comparable to the current report; other studies may not have had the statistical power to identify *SLCO1B1* at the genome-wide level.

Along with *LPA*, *APOE*, and *SLCO1B1*, Postmus et al. also reported *SORT1* as a genome-wide predictor of statin LDL-C response.^15^ However, a baseline-adjusted phenotype was used to perform this GWAS. Of note, though the inclusion of baseline in the regression increases the chances of false-positives, it does not warrant a complete dismissal of all results generated with this approach (some of which may be true positives). That being stated, there are multiple lines of evidence suggesting that *SORT1* may not be a true genetic predictor of statin-induced LDL-C change. First, in a post-hoc interaction study, Postmus et al. demonstrated that among their four genome-wide significant hits, while *LPA*, *APOE*, and *SLCO1B1* showed a significant interaction (or suggestive interaction) with statin effect at the genome-wide level, the interaction *P* value for the *SORT1* hit was far from genome-wide significant (*P* = 0.047). Second, our interaction results of the current study were similar to the above interaction results (top hit for *SORT1* [rs7528419] interaction *P* value = 0.009). Third, Postmus et al. showed that while adjusting for measurement error at *LPA*, *APOE*, and *SLCO1B1* only modestly attenuated the apparent genetic effect with statin LDL-C response, the measurement error-adjusted effect for *SORT1* was dramatically attenuated (by 65 to 68%). And finally our own baseline-adjusted results of the current study replicated all 4 hits reported by Postmus et al. providing a positive control that *SORT1* is only associated with LDL-C response to statins when baseline LDL-C levels are inappropriately added to the model as a covariate. Further studies are necessary investigating the role of *SORT1* variants in statin-induced LDL-C lowering.

To assess how commonly baseline adjustment had been conducted in pharmacogenomic studies, we systematically reviewed GWASs of drug-induced quantitative change. We determined that the majority of studies performed flawed baseline-adjusted analyses. Furthermore, we found that most of the studies were published in recent years. Clearly, this type of error is widespread in the pharmacogenomics community and persists today, though it is unknown how many false-positive associations have been disseminated due to this practice. As the use of quantitative biomarkers for drug efficacy and safety continues to grow in pharmacogenomics research, it is important that this erroneous approach is avoided in future studies to prevent any further reporting of false-positive genetic associations in the literature.

Approaches beyond using baseline as a regression covariate include matching, stratifying, and excluding patients by baseline value. These methods generate the same bias as the baseline covariate.^3^ Another approach is to use the baseline-adjusted on-treatment level alone (rather than quantitative change) as the response variable. This method has high statistical power and performs well in randomized controlled trials, but induces large biases with more heterogeneous observational data.^17^ We confirmed this bias in a GWAS of statin on-treatment LDL-C adjusted for baseline LDL-C (Supplementary Table 8), which also produced false-positive genome-wide hits similar to other baseline-adjusted analyses in the current report. One may also adjust for baseline measurement error variance.^2^ However, this approach requires an estimate of measurement error variance (e.g., through repeat measures), which many not be feasible to do. Furthermore, the impact of regression to the mean may not be adequately removed through this approach.^18^

These consequences of baseline-adjusted analyses also apply to the pharmacogenetic candidate gene approach with phenotypes of quantitative change. This is because a common method in candidate gene studies is to compare mean change between groups defined by genotype or haplotype using analysis of covariance (ANCOVA) to adjust for baseline. Past studies in statistics show that the “regression toward the mean” principle described in this current work occurs for ANCOVA.^19,20^

Evidently, no options are free of limitations. We generally recommend that researchers performing pharmacogenomic studies of quantitative change observe the following best practices: (a) use multiple baseline measures if available to reduced measurement error, (b) perform baseline-unadjusted analyses on change response variables, and (c) perform interaction analyses (e.g., baseline vs. on-treatment) to confirm the true genetic effects on drug response.

Our results support that of not adjusting for baseline in GWASs of quantitative change. A concerted effort from the scientific community to implement appropriate statistical approaches in pharmacogenomic studies will improve the reporting of true genetic determinants of drug response in the literature and ultimately improve the translation of pharmacogenomic discovery into clinical application.

## Methods

### Data source and study population

We used electronic health records (EHRs) from the Kaiser Permanente Genetic Epidemiology Research on Adult Health and Aging (GERA) cohort as previously described.^21–23^ Lipid panel measurements, statin dispensing records, and other medical records from individuals who initiated statin therapy between 1996 and early 2018 were extracted for the study. We used the same methods as previously described to define a new statin user and classify individuals by cardiovascular risk factor status (hypertension, diabetes, cigarette smoking status).^23^ Briefly, participants had to have ≥2 dispensing records of a statin (to exclude potentially nonadherent participants) as well as ≥1 lipid measurement before and after statin initiation (to determine statin LDL-C response). Approval was obtained from Kaiser Permanente and University of California Institutional Review Boards. Participants gave written informed consent.

### Phenotype

We used a pair of lipid measurements from each participant: the most recent pretreatment level (designated as the baseline) and earliest on-treatment LDL-C from the EHRs before and after statin initiation, respectively. On-treatment LDL-C values had to have been measured within a time frame starting 3 weeks after statin initiation and ending 3 weeks after the initial statin fill days’ supply. Lipid measurements within the time frame of a non-statin LDL-C therapy (bile acid sequestrants, ezetimibe, fibrates, prescription niacin, and prescription omega-3 fatty acids) were excluded. All lipid measurement data collected from the EHRs were fasting levels, were obtained in the course of patient care, and were assayed in Kaiser Permanente laboratories, as previously described.^10,23^ From the pair of LDL-C measurements per participant, we calculated statin LDL-C response using two formulas. The first formula was based on the definition of Postmus et al.^15^ as the difference between the natural log-transformed baseline LDL-C (X) and on-treatment LDL-C (Y) values adjusted for the natural log-transformed baseline value: ln(Y) − ln(X) adjusted for ln(X). The second formula was a percent change in LDL-C from statin therapy that we used previously.^23^ Briefly, response was expressed as (Y − X)/X. Both phenotypes were adjusted for the following prespecified covariates (at the time of statin initiation) within self-reported race/ethnicity groups for each participant: age, sex, body mass index (BMI), statin type, statin dose, hypertension, diabetes, and cigarette smoking (current/former). Each race/ethnicity group (White/European, Black/African, East Asian, and Hispanic/Latino) was analyzed separately. Participants who did not self-identify within these four race/ethnicity groups were excluded. Statin dose adjustment was based on a revised defined daily dose equivalency table as previously described.^23^ Genetic ancestry eigenvectors previously generated from principal component analyses within the race/ethnicity groups were also included as covariates.^21^ In particular, the first ten eigenvectors for White/Europeans and the first six eigenvectors for Black/Africans, East Asians, and Hispanic/Latinos were included as covariates. The additional eigenvectors in White/Europeans were to ensure robustness against any potential minute population structure variation that might be detected within this larger race/ethnicity group. To investigate the impact of adjusting for baseline LDL-C values, both calculations of statin LDL-C response were performed with and without baseline adjustment. This resulted in four phenotypes (regression models) within each race/ethnicity group for interrogation of genome-wide variants: ln(Y) − ln(X) adjusted for ln(X), (Y − X)/X, ln(Y) − ln(X), and (Y − X)/X adjusted for X. Residuals were rank-normalized for the two phenotypes that were not already transformed: (Y − X)/X and (Y − X)/X adjusted for X.

### Genotype

Study participants were previously genotyped on one of four Affymetrix Axiom arrays (ranging from 674,518 to 893,631 variants) based on self-reported race/ethnicity.^21,22,24^ Imputation was performed to the 1000 Genomes Project (Phase I integrated release, March 2012, with August 2012 chromosome update),^10^ as has been described. Only common variants were included in the analysis. For Black/Africans, East Asians, and Hispanic/Latinos, minor allele frequencies >1% were considered common variants. Due to a larger sample size, a more liberal minor allele frequency threshold (>0.05%) was used to define common variants in White/Europeans. These minor allele frequency thresholds ensured a minimum minor allele count of ten within each race/ethnicity group.

### Statistical analysis

Residuals derived from the above regression models (four response formulas for each of four race/ethnicity groups) were evaluated as a function of genotype using multiple linear regression in an additive model of inheritance. For imputed variants, the phenotypes were evaluated as a function of allelic dosage.^25^ A fixed-effects meta-analysis of the combined race/ethnicity groups was then conducted to generate the final genome-wide association results (for each of the four phenotypes). We also conducted repeat GWASs with untransformed residuals for the (Y − X)/X phenotype to approximate an effect size (beta) of the associations in the units of percent change from baseline. Finally, we performed an interaction analysis to determine the effect of statin on baseline versus on-treatment LDL-C values. For this, we first ran regression analyses on the natural log-transformed baseline and on-treatment LDL-C values to generate residuals for ln(X) and ln(Y), respectively. For ln(Y), we adjusted for the following covariates analogous to the statin LDL-C response phenotypes described above: age, sex, BMI, statin type, statin dose, hypertension, diabetes, cigarette smoking (current/former) and genetic ancestry eigenvectors in population-stratified analyses. A similar regression model was used for ln(X) except statin type and statin dose were not added to the model. The beta of each variant was then generated for baseline and on-treatment LDL-C values by conducting GWAS of the ln(X) and ln(Y) residuals, respectively. A Cochran’s Q test (genome-wide heterogeneity test) comparing baseline versus on-treatment betas was performed to test the gene−drug interaction of each variant. For all genome-wide analyses, *P* < 5 × 10^−8^ was considered to meet genome-wide significance and *P* < 1 × 10^−5^ was considered to be suggestive of an association. Significant/suggestive variants >0.5 Mb from each other were considered to be from independent loci. Statistical analyses were conducted with R (R Foundation for Statistical Computing, version 3.5.1, https://www.R-project.org/) and PLINK (version 1.07, http://pngu.mgh.harvard.edu/purcell/plink/).^26^ All statistical tests were two-sided.

### Literature search

We conducted a comprehensive review of previous GWASs from an online resource maintained by the National Human Genome Research Institute (NHGRI-EBI GWAS Catalog; accessed 02/07/19)^27^ and determined how often drug response studies included quantitative change phenotypes that adjusted for baseline. We accomplished this in three stages. In stage 1, we used four search terms (“pharm”, “drug”, “treat”, “response”) to select for GWASs of drug response phenotypes. Studies that included ≥1 of these search terms in its title or disease/trait description were carried forward for further review. In stage 2, the studies selected from stage 1 were manually reviewed to: (1) confirm that the studies included a phenotype of a pharmacologic intervention in humans, (2) select only studies that included ≥1 phenotype(s) of change in a quantitative measurement (as a response to a pharmacologic intervention), and (3) select only studies that included clinical or demographic covariates in the linear regression model. Phenotypes of vaccine effects, antibody titer response, drug-treated cell lines, and pharmacokinetic metrics were not considered phenotypes of drug response for the purposes of this study. We also excluded drug−gene interaction studies not involving quantitative change phenotypes (i.e., baseline versus on-treatment measurement values), abstracts, and sensitivity analyses of an exploratory nature. Moreover, studies using the ANCOVA approach were inherently excluded, as GWASs of quantitative change are primarily regression analyses. In stage 3, among the final set of studies selected from stage 2, we determined the proportion of studies that included baseline as a covariate in the regression model.

### Reporting summary

Further information on research design is available in the Nature Research Reporting Summary linked to this article.

## Supplementary information

Supplementary Information

Reporting Summary

## Data Availability

Genotype data are available on approximately 78% of GERA participants from the database of Genotypes and Phenotypes (dbGaP) under accession phs000674. v1.p1. This includes individuals who consented to having their data shared with dbGaP. The complete GERA data are available upon application to the Kaiser Permanente Research Bank Portal (https://researchbank.kaiserpermanente.org/for-researchers/). Summary statistics will be made publicly available from the National Human Genome Research Institute-European Bioinformatics Institute (NHGRI-EBI) GWAS Catalog, (https://www.ebi.ac.uk/gwas/downloads/summary-statistics).
