# Supplementary material for: The impact of adjusting for baseline in pharmacogenomic genome-wide association studies of quantitative change
Source: NPJ Genom Med. 2020 Jan 16;5:1. doi: 10.1038/s41525-019-0109-4 (PMC6965183; doi:10.1038/s41525-019-0109-4)
Supplement: Supplementary file 1 — Supplementary Information [file 41525_2019_109_MOESM1_ESM.pdf]

Supplemental data

Supplemental Figure 1.

Manhattan plot for a genome wide association study (GWAS) of baseline low-density lipoprotein cholesterol (LDL-C) levels

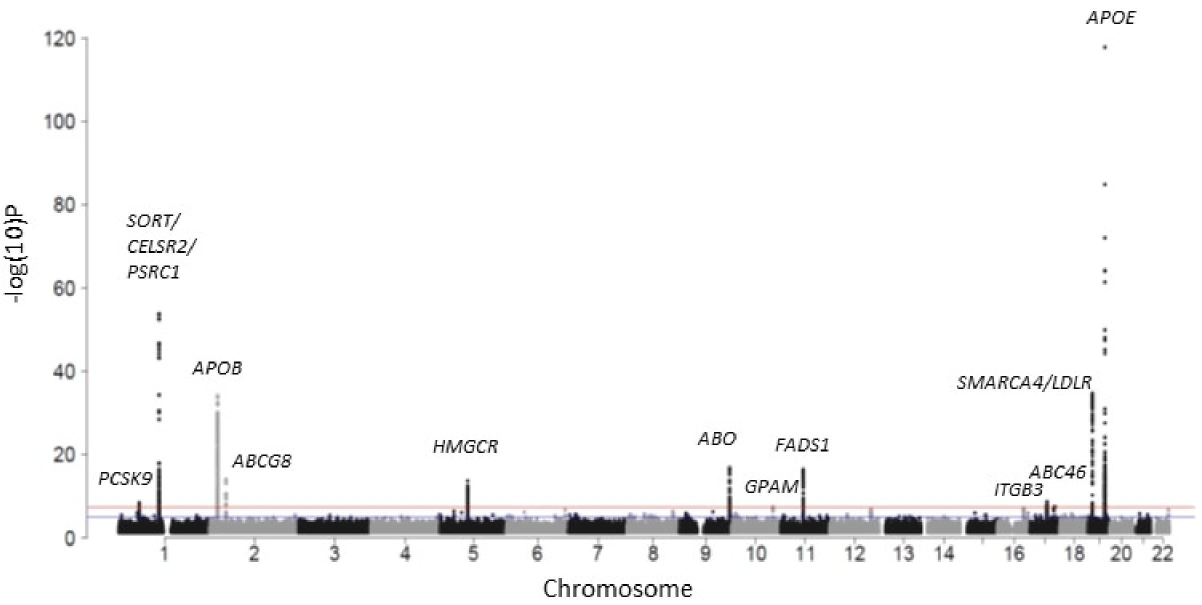

A GWAS of natural log-transformed baseline LDL-C levels from a fixed-effects meta-analysis of combined race/ethnicity groups (White/European, Black/African, East Asian, and Hispanic/Latino) yielded multiple significant loci. P values were generated using linear regression analysis adjusted for age, sex, BMI, hypertension, diabetes, cigarette smoking, and genetic ancestry eigenvectors in population-stratified analyses. All tests were two-sided.

Supplemental Figure 2.

Manhattan plot for a genome wide association study (GWAS) of statin on-treatment low-density lipoprotein cholesterol (LDL-C) levels

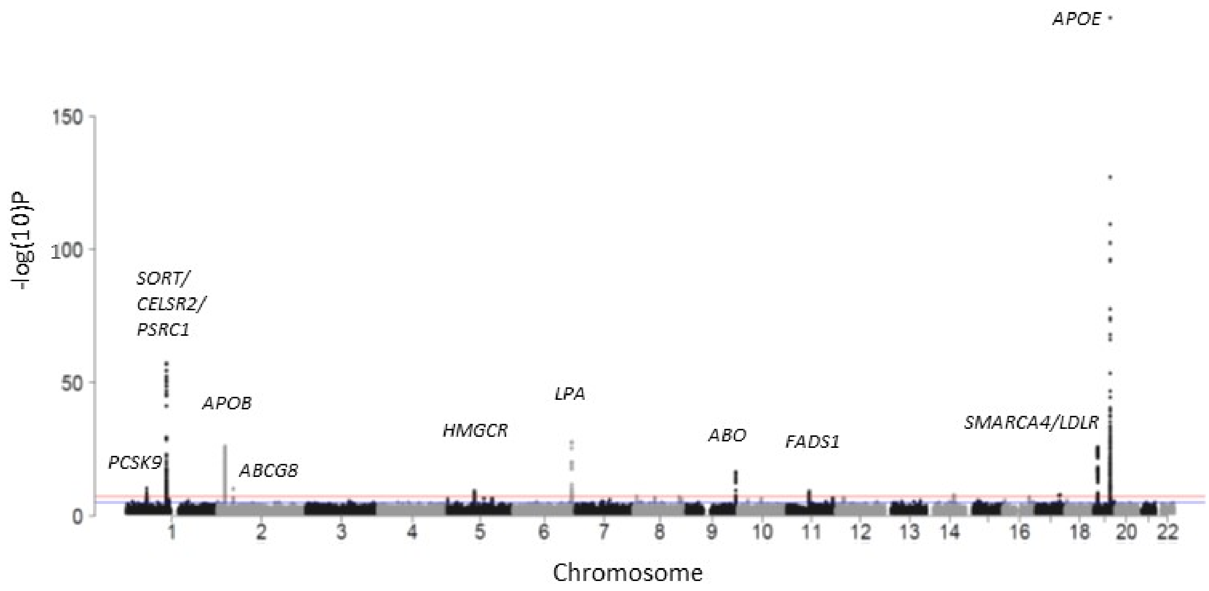

A GWAS of natural log-transformed statin on-treatment LDL-C levels from a fixed-effects meta-analysis of combined race/ethnicity groups (White/European, Black/African, East Asian, and Hispanic/Latino) yielded multiple significant loci. P values were generated using linear regression analysis adjusted for age, sex, BMI, statin type, statin dose, hypertension, diabetes, cigarette smoking, and genetic ancestry eigenvectors in population-stratified analyses. All tests were two-sided.

Supplemental Table 1.

Descriptive factors of the study population at the time of statin initiation overall and by race/ethnicity[1]

	White/European	Black/African	East Asian	Hispanic/Latino	Total
Sample size	28,616	1,205	2,350	2,703	34,874
Age (years)	65 (58 to 72)	62 (55 to 68)	61 (54 to 69)	62 (54 to 69)	65 (58 to 72)
Female	15,116 (53%)	687 (57%)	1,146 (49%)	1,397 (52%)	18,346 (53%)
Body mass index (kg/m^2)	27.3 (24.3 to 30.9)	29.6 (26.0 to 34.0)	25.1 (22.9 to 27.8)	28.6 (25.4 to 32.5)	27.3 (24.3 to 30.9)
Hypertension	16,469 (58%)	929 (77%)	1,411 (60%)	1,520 (56%)	20,329 (58%)
Diabetes	4649 (16%)	412 (34%)	559 (24%)	845 (31%)	6,465 (19%)
Cigarette smoking[2]	14,391 (50%)	608 (50%)	804 (34%)	1,217 (45%)	17020 (49%)
Statin type					
Simvastatin	8,825 (31%)	844 (31%)	296 (25%)	844 (31%)	10,655 (31%)
Lovastatin	17,379 (61%)	1,646 (61%)	823 (68%)	1,646 (61%)	21,302 (61%)
Atorvastatin	2,022 (7%)	176 (7%)	72 (6%)	176 (7%)	2,443 (7%)
Pravastatin	388 (1%)	37 (1%)	14 (1%)	37 (1%)	472 (1%)
Statin log2(DDD)[3]	-0.47 (1.06)	-0.55 (1.06)	-0.534 (1.05)	-0.44 (1.04)	-0.48 (1.05)
Lipid panel (mg/dL)					
LDL-C	152 (129 to 174)	148 (123 to 173)	151 (127 to 179)	148 (123 to 173)	152 (128 to 174)
HDL-C	51 (43 to 62)	48 (41 to 57)	52 (44 to 62)	48 (41 to 57)	51 (43 to 62)
Total cholesterol	238 (209 to 264)	234 (204 to 262)	232 (202 to 262)	234 (204 to 262)	238 (209 to 264)
Triglycerides	140 (100 to 197)	156 (114 to 215)	115 (87 to 155)	156 (114 to 215)	141 (101 to 197)
Treated LDL-C (mg/dL)[4]	98 (79 to 119)	100 (80 to 125)	95 (77- to 116)	96 (75 to 118)	98 (78 to 119)
LDL-C lowering (%)[45]	-35 (-45 to -24)	-34 (-44 to -22)	-36 (-46 to -24)	-35 (-45 to -23)	-34 (-45 to -24)

HDL-C, high-density lipoprotein cholesterol; LDL-C; low-density lipoprotein cholesterol. Data are shown as median (interquartile range) or count (%) unless otherwise stated

[1]Race/ethnicity was determined by self-report

[2]Current or former cigarette smoking

[3]The binary logarithm of statin defined daily dose. A unit of 0 is equivalent to atorvastatin 10mg, lovastatin 40mg, pravastatin 40mg, and simvastatin 20mg daily. Data are show as mean (standard deviation). A negative value indicates weaker doses

[4]Following statin initiation

[5]A negative value indicates more intense statin LDL-C lowering.

Supplemental Table 2.

All six lead variants from the genome-wide significant meta-analysis loci for baseline-adjusted difference of natural log-transformed statin on- and baseline low-density lipoprotein cholesterol levels (LDL-C) by statin response variable in White/Europeans (n=28,616)

Gene	SNP	CHR	BP	Minor allele	MAF	Statin response variable[1]	Beta (SE)[2]	P-value
SORT1	rs7528419	1	109817192	G	0.210	Baseline-adjusted	-0.019 (0.002)	8.26E-16
						Baseline-unadjusted	-0.045 (0.011)	2.93E-05
						Baseline only	-0.036 (0.002)	1.24E-47
APOB	rs1713222	2	21271323	A	0.154	Baseline-adjusted	-0.012 (0.003)	2.12E-06
						Baseline-unadjusted	-0.025 (0.012)	3.22E-02
						Baseline only	-0.027 (0.003)	3.70E-24
LPA	rs10455872	6	161010118	G	0.072	Baseline-adjusted	0.041 (0.004)	1.88E-29
						Baseline-unadjusted	0.171 (0.016)	7.02E-26
						Baseline only	0.009 (0.004)	0.013
SLC01B1	rs58310495	12	21357711	T	0.157	Baseline-adjusted	0.015 (0.003)	2.21E-09
						Baseline-unadjusted	0.069 (0.012)	1.91E-09
						Baseline only	-0.0002 (0.003)	0.943
SMARCA4/LDLR	rs67337506	19	11207982	C	0.219	Baseline-adjusted	0.011 (0.003)	8.69E-06
						Baseline-unadjusted	-0.026 (0.011)	2.27E-02
						Baseline only	-0.019 (0.003)	2.37E-13
APOE	rs7412	19	45412079	T	0.071	Baseline-adjusted	0.070 (0.004)	1.15E-75

						Baseline-unadjusted	-0.211 (0.017)	1.29E-35
						Baseline only	-0.083 (0.004)	6.45E-103

BP, base pair; CHR, chromosome; MAF, minor allele frequency; SE, standard error; SNP, single nucleotide polymorphism

[1]Baseline-adjusted difference of natural log-transformed statin on- and baseline low-density lipoprotein cholesterol levels, baseline-unadjusted statin-induced percent low-density lipoprotein cholesterol lowering, and natural log-transformed baseline low-density lipoprotein cholesterol level

[2] Fixed effects calculated with respect to the minor allele. A negative value indicates more intense statin LDL-C lowering.

Supplemental Table 3.

<u>All six lead variants from the genome-wide significant meta-analysis loci for baseline-adjusted difference of natural log-transformed statin on- and baseline low-density lipoprotein cholesterol (LDL-C) levels by statin response variable in Black/Africans (n=1,205)</u>

Gene	SNP	CHR	BP	Minor allele	MAF	Statin response variable[1]	Beta (SE)[2]	P-value
SORT1	rs7528419	1	109817192	G	0.242	Baseline-adjusted	-0.024 (0.011)	0.034
						Baseline-unadjusted	-0.094 (0.049)	0.055
						Baseline only	-0.035 (0.012)	0.004
APOB	rs1713222	2	21271323	A	0.256	Baseline-adjusted	-0.012 (0.011)	0.287
						Baseline-unadjusted	-0.038 (0.047)	0.427
						Baseline only	-0.043 (0.012)	0.004
LPA	rs10455872	6	161010118	G	0.025	Baseline-adjusted	-0.038 (0.034)	0.269
						Baseline-unadjusted	0.217 (0.148)	0.142
						Baseline only	-0.005 (0.037)	0.889
SLC01B1	rs58310495	12	21357711	T	0.173	Baseline-adjusted	0.016 (0.013)	0.205
						Baseline-unadjusted	0.061 (0.055)	0.266
						Baseline only	0.005 (0.014)	0.707
SMARCA4/LDLR	rs67337506	19	11207982	T	0.432	Baseline-adjusted	0.010 (0.010)	0.315
						Baseline-unadjusted	0.049 (0.041)	0.234
						Baseline only	0.008 (0.010)	0.455
APOE	rs7412	19	45412079	T	0.093	Baseline-adjusted	-0.047 (0.016)	0.004

						Baseline-unadjusted	-0.117 (0.071)	0.098
						Baseline only	-0.102 (0.017)	4.93E-09

BP, base pair; CHR, chromosome; MAF, minor allele frequency; SE, standard error; SNP, single nucleotide polymorphism

[1]Baseline-adjusted difference of natural log-transformed statin on- and baseline low-density lipoprotein cholesterol levels, baseline-unadjusted statin-induced percent low-density lipoprotein cholesterol lowering, and natural log-transformed baseline low-density lipoprotein cholesterol level

[2]Fixed effects calculated with respect to the minor allele. A negative value indicates more intense statin LDL-C lowering.

Supplemental Table 4.

All six lead variants from the genome-wide significant meta-analysis loci of for baseline-adjusted difference of natural log-transformed statin on- and baseline low-density lipoprotein cholesterol levels (LDL-C) by statin response variable in East Asians (n=2,350)

Gene	SNP	CHR	BP	Minor allele	MAF	Statin response variable[1]	Beta (SE)[2]	P-value
SORT1	rs7528419	1	109817192	G	0.059	Baseline-adjusted	-0.019 (0.015)	0.232
						Baseline-unadjusted	-0.101 (0.066)	0.126
						Baseline only	-0.007 (0.016)	0.662
APOB	rs1713222	2	21271323	A	0.016	Baseline-adjusted	0.006 (0.028)	0.831
						Baseline-unadjusted	-0.014 (0.118)	0.904
						Baseline only	0.004 (0.028)	0.898
LPA	rs56393506	6	161089307	T	0.122	Baseline-adjusted	0.017 (0.011)	0.134
						Baseline-unadjusted	0.066 (0.047)	0.161
						Baseline only	0.001 (0.011)	0.916
SLC01B1	rs58310495	12	21357711	T	0.439	Baseline-adjusted	0.006 (0.007)	0.343
						Baseline-unadjusted	0.037 (0.029)	0.208
						Baseline only	-0.002 (0.007)	0.740
SMARCA4/LDLR	rs67337506	19	11207982	C	0.426	Baseline-adjusted	-0.015 (0.008)	0.079
						Baseline-unadjusted	-0.052 (0.036)	0.148
						Baseline only	-0.001 (0.009)	0.933
APOE	rs141622900	19	45426792	A	0.016	Baseline-adjusted	-0.060 (0.042)	0.150

						Baseline-unadjusted	-0.204 (0.178)	0.252
						Baseline only	-0.041 (0.042)	0.332

BP, base pair; CHR, chromosome; MAF, minor allele frequency; SE, standard error; SNP, single nucleotide polymorphism

[1]Baseline-adjusted difference of natural log-transformed statin on- and baseline low-density lipoprotein cholesterol levels, baseline-unadjusted statin-induced percent low-density lipoprotein cholesterol lowering, and natural log-transformed baseline low-density lipoprotein cholesterol level

[2]Fixed effects calculated with respect to the minor allele. A negative value indicates more intense statin LDL-C lowering.

Supplemental Table 5.

<u>All six lead variants from the genome-wide significant meta-analysis loci for baseline-adjusted difference of natural log-transformed statin on- and baseline low-density lipoprotein cholesterol levels (LDL-C) by statin response variable in Hispanic/Latinos (n=2,703)</u>

Gene	SNP	CHR	BP	Minor allele	MAF	Statin response variable[1]	Beta (SE)[2]	P-value
SORT1	rs7528419	1	109817192	G	0.190	Baseline-adjusted	-0.016 (0.009)	0.069
						Baseline-unadjusted	-0.016 (0.035)	0.640
						Baseline only	-0.035 (0.009)	4.40E-05
APOB	rs1713222	2	21271323	A	0.132	Baseline-adjusted	-0.031 (0.010)	0.001
						Baseline-unadjusted	-0.092 (0.040)	0.022
						Baseline only	-0.027 (0.010)	0.006
LPA	rs10455872	6	161010118	G	0.043	Baseline-adjusted	0.075 (0.016)	4.86E-06
						Baseline-unadjusted	0.301 (0.067)	7.01E-06
						Baseline only	-0.007 (0.017)	0.676
SLC01B1	rs58310495	12	21357711	T	0.145	Baseline-adjusted	0.031 (0.009)	0.001
						Baseline-unadjusted	0.134 (0.039)	5.12E-04
						Baseline only	-0.015 (0.010)	0.122
SMARCA4/LDLR	rs67337506	19	11207982	C	0.269	Baseline-adjusted	-0.021 (0.007)	0.004
						Baseline-unadjusted	-0.063 (0.031)	0.038
						Baseline only	-0.020 (0.008)	0.010
APOE	rs7412	19	45412079	T	0.040	Baseline-adjusted	-0.059 (0.017)	5.01E-04

						Baseline-unadjusted	-0.126 (0.070)	0.073
						Baseline only	-0.121 (0.017)	3.11E-12

BP, base pair; CHR, chromosome; MAF, minor allele frequency; SE, standard error; SNP, single nucleotide polymorphism

[1]Baseline-adjusted difference of natural log-transformed statin on- and baseline low-density lipoprotein cholesterol levels, baseline-unadjusted statin-induced percent low-density lipoprotein cholesterol lowering, and natural log-transformed baseline low-density lipoprotein cholesterol level

[2]Fixed effects calculated with respect to the minor allele. A negative value indicates more intense statin LDL-C lowering.

Supplemental Table 6.

Genome wide association studies (GWAS) with quantitative phenotypes of drug response published through 2018

First author	Publication year	Phenotype		Adjusted for baseline?
		Quantitative measure change	Drug	
Das et al.	2018	High-density lipoprotein cholesterol, triglyceride	Fenofibrate	No
Massey et al.	2018	Erythrocyte sedimentation rate (rheumatoid arthritis)	Tumor necrosis factor inhibitor	Yes
Lorés-Motta et al.	2018	Visual acuity in neovascular age-related macular degeneration	Anti-vascular endothelial growth factor therapy (bevacizumab or ranibizumab)	Yes
Li et al.	2018	Positive and Negative Syndrome Scale (schizophrenia)	Lurasidone	No
Rotroff et al.	2018	Hemoglobin A1c (type 2 diabetes)	Metformin	Yes
Ala-Mutka et al.	2018	Serum uric acid in hypertension	Hydrochlorothiazide	Yes
Singh et al.	2018	Glucose in hypertension	Thiazide diuretic	Yes
Yu et al.	2018	Positive and Negative Syndrome Scale (schizophrenia)	Olanzapine	No
Shahin et al.	2018	Heart rate	Atenolol	Yes
Inaba et al.	2018	Bone mineral density change in acute lymphoblastic leukemia	Combined chemotherapy treatment	Yes
Rotroff et al.	2018	Triglyceride in statin-treated type 2 diabetes	Fenofibrate	Yes
Maciukiewicz et al.	2018	Montgomery-Åsberg Depression Rating Scale score (major depressive disorder)	Duloxetine	No
Li et al.	2017	Positive Marder score (schizophrenia)	Paliperidone	Yes

Salvi et al.	2017	Diastolic blood pressure (hypertension)	Hydrochlorothiazide	Yes
Mosteller et al.	2017	Forced expiratory volume (asthma)	Inhaled corticosteroid	Yes
Magvanjav et al.	2017	Systolic blood pressure, diastolic blood pressure (hypertension)	Combination thiazide diuretic and beta-blocker	Yes
Yamashiro et al.	2017	Visual acuity in neovascular age-related macular degeneration	Ranibizumab	No
Serie et al.	2017	Left ventricular ejection fraction in HER2-positive breast cancer	Paclitaxel	Yes
Yeo et al.	2017	Lipoprotein-associated phospholipase A2 activity in cardiovascular disease	Darapladib	Yes
Wells et al.	2017	Left ventricular function	Anthracycline	Yes
Hardin et al.	2016	Forced expiratory volume (chronic obstructive pulmonary disease)	Bronchodilator	No
Postmus et al.	2016	High-density lipoprotein cholesterol	Statin	Yes
Zhou et al.	2016	Hemoglobin A1c (type 2 diabetes)	Metformin	Yes
Irvin et al.	2016	High-density lipoprotein cholesterol	Fenofibrate	Yes
Yu et al.	2016	Weight gain in schizophrenia	Atypical antipsychotic	No
Brandl et al.	2016	Weight gain in schizophrenia	Atypical antipsychotic	Yes
Dahlin et al.	2016	Forced expiratory volume (asthma)	Montelukast	Yes
Dahlin et al.	2016	Forced expiratory volume (asthma)	Zileuton	Yes
Wen et al.	2015	Serum uric acid (gout)	Allopurinol	Yes
Shim et al.	2015	Low-density lipoprotein cholesterol subfraction	Statin	No

Jeong et al.	2015	Intraocular pressure	Triamcinolone acetonide	No
Israel et al.	2015	Forced expiratory volume (asthma)	Inhaled β2-agonist	No
Chittani et al.	2015	Systolic blood pressure	Hydrochlorothiazide	Yes
Del-Aguila et al.	2015	Potassium (hypokalemia)	Hydrochlorothiazide	Yes
Postmus et al.	2014	Low-density lipoprotein cholesterol	Statin	Yes
Theusch et al.	2014	Proprotein convertase subtilisin/kexin type 9 protein	Simvastatin	No
Frau et al.	2014	Systolic blood pressure	Losartan	Yes
Park et al.	2014	Forced expiratory volume (asthma)	Inhaled corticosteroid	No
Duan et al.	2014	Forced expiratory volume (asthma)	Inhaled β2-agonist	Yes
Del-Aguila et al.	2014	Fasting plasma glucose, triglycerides in hypertension	Hydrochlorothiazide	Yes
Kamide et al.	2013	Systolic blood pressure, diastolic blood pressure (hypertension)	Anti-hypertensive	Yes
Turner et al.	2013	Systolic blood pressure, diastolic blood pressure (hypertension)	Hydrochlorothiazide	Yes
Clark et al.	2013	Global impression of severity scale (schizophrenia)	Antipsychotic	No
Aslibekyan et al.	2013	Adiponectin	Fenofibrate	No
Hopewell et al.	2013	Low-density lipoprotein cholesterol, apolipoprotein B	Simvastatin	No
Chu et al.	2012	Lipoprotein-associated phospholipase A2 activity	Rosuvastatin	No
Frazier-Wood et al.	2012	Fasting lipid particle diameter	Fenofibrate	No
Himes et al.	2012	Forced expiratory volume (asthma)	Inhaled β2-agonist	No

Malhotra et al.	2012	Weight gain in schizophrenia	Antipsychotic	Yes
Deshmukh et al.	2012	Low-density lipoprotein cholesterol	Statin	Yes
Aslibekyan et al.	2012	Inflammatory biomarker pattern	Fenofibrate	No
Aberg et al.	2012	QTc interval prolongation	Antipsychotic	No
Tantisira et al.	2011	Forced expiratory volume (asthma)	Inhaled corticosteroid	No
Mick et al.	2011	Systolic blood pressure, diastolic blood pressure	Methylphenidate	No
Adkins et al.	2011	Weight, blood lipids, glucose and hemoglobin A1c, blood pressure and heart rate	Antipsychotic	No
Barber et al.	2010	Cholesterol, triglyceride	Statin	No
Ge et al.	2009	Viral clearance	Hepatitis C treatment	Yes
Thompson et al.	2009	Low-density lipoprotein cholesterol, high-density lipoprotein cholesterol, or triglyceride	Atorvastatin	Yes
Volpi et al.	2009	QT prolongation	Iloperidone	Yes

Supplemental Table 7.

The impact of statin-induced absolute low-density lipoprotein cholesterol (LDL-C) reduction (milligrams per deciliter) with all six lead variant from the genome-wide significant meta-analysis loci for baseline-adjusted difference of natural log-transformed statin on- and baseline low-density lipoprotein cholesterol levels in combined race/ethnicity groups (N=34,874)

Gene	SNP	CHR	BP	Minor allele	MAF	Statin response variable[1]	Beta (SE)[2]	P-value	Cochrane's Q statistic p-value[3]	I^2 heterogeneity index (0-100) [3]
SORT1	rs7528419	1	109817192	G	0.198	Difference of logs	-0.019 (0.002)	9.55E-18	0.944	0
						Absolute reduction	-1.664	1.50E-14	0.919	0
APOB	rs1713222	2	21271323	A	0.145	Difference of logs	-0.013 (0.002)	4.68E-08	0.255	26
						Absolute reduction	-1.202	3.86E-07	0.502	0
LPA	rs10455872	6	161010118	G	0.068	Difference of logs	0.042 (0.003)	1.01E-33	0.128	51
						Absolute reduction	3.880	3.54E-30	0.198	38
SLC01B1	rs58310495	12	21357711	T	0.179	Difference of logs	0.015 (0.002)	4.58E-11	0.225	31
						Absolute reduction	1.451	4.70E-11	0.301	18
SMARCA4/LDLR	rs67337506	19	11207982	C	0.250	Difference of logs	-0.012 (0.002)	3.08E-08	0.602	0
						Absolute reduction	-1.109	2.67E-07	0.508	0
APOE	rs7412	19	45412079	T	0.070	Difference of logs	-0.068 (0.004)	1.08E-78	0.35	5
						Absolute reduction	-5.570	1.82E-57	0.678	0

BP, base pair; CHR, chromosome; MAF, minor allele frequency; SE, standard error; SNP, single nucleotide polymorphism

[1]Baseline-adjusted difference of natural log-transformed statin on- and baseline low-density lipoprotein cholesterol levels and baseline-adjusted statin-induced absolute low-density lipoprotein cholesterol lowering

[2]Fixed effects calculated with respect to the minor allele. A negative value indicates more intense statin LDL-C lowering.

[3]Refers to the variation on results between race/ethnicity groups

Supplemental Table 8.

Lead variants from genome-wide significant loci of baseline-adjusted natural log-transformed statin on-treatment low-density lipoprotein cholesterol (LDL-C) levels in combined and individual race/ethnicity groups

Race/ethnicity group	Gene	SNP	CHR	BP	Minor allele	MAF	Beta (SE)[1]	P-value
Combined	*SORT1*	rs646776	1	109818530	C	0.203	-0.020 (0.002)	2.93E-16
	LPA	rs10455872	6	161010118	G	0.068	0.042 (0.003)	2.05E-34
	SLC01B1	rs58310495	12	21357711	T	0.179	0.016 (0.002)	1.80E-11
	APOE	rs7412	19	45412079	T	0.070	-0.078 (0.004)	1.01E-89
White/Europeans	*PCSK9*	rs11591147	1	55505647	T	0.011	-0.073 (0.012)	8.05E-10
	Unknown	rs150561664	1	69876849	A	0.001	-0.465 (0.079)	3.73E-09
	SORT1	rs646776	1	109818530	C	0.210	-0.020 (0.002)	2.93E-16
	Unknown	rs2851341	4	109641691	A	0.001	-0.351 (0.062)	1.68E-08
	LPA	rs55730499	6	161005610	T	0.073	0.041 (0.004)	8.22E-30
	SLC01B1	rs1871395	12	21352315	G	0.157	0.016 (0.003)	4.32E-10
	APOE	rs7412	19	45412079	T	0.071	-0.074 (0.004)	1.53E-84
	Unknown	rs5966882	23	98005912	C	0.008	-0.080 (0.014)	5.04E-09

BP, base pair; CHR, chromosome; MAF, minor allele frequency; SE, standard error; SNP, single nucleotide polymorphism

[1]Fixed effects calculated with respect to the minor allele. A negative value indicates more intense statin LDL-C lowering.
